# Are current avian influenza vaccines a solution for smallholder poultry farmers?

**DOI:** 10.12688/gatesopenres.13171.1

**Published:** 2020-08-26

**Authors:** Vincent Guyonnet, Andew R. Peters

**Affiliations:** 1 FFI Consulting Ltd., Brockville, Ontario, Canada; 2 Supporting Evidence Based Interventions (SEBI), University of Edinburgh, Edinburgh, UK

**Keywords:** Avian Influenza, vaccines, vaccination, poultry smallholders

## Abstract

Vaccination against highly pathogenic avian influenza (HPAI) viruses, along with other measures, was successful in eradicating AI in very few countries where the competence of national veterinary services or the geography and bird density have contributed favorably to the outcome.  The main constraints to an effective AI vaccination are vaccine composition matching field strains, reliable cold chain and logistics to target all poultry smallholders, constraints related to the availability of sufficient financial and human resources.  When not conducted properly, vaccination can also contribute to the emergence of new field viral strains, through genetic drifts of HPAI viruses.   While new technologies have improved the possibility to produce high quality vaccines matching field strains, recurrent issues like post-vaccination field surveillance and vaccination coverage continue to limit the relevance of AI vaccination in smallholder settings. A “game-changer” vaccine targeting smallholders should be universal to protect against all field viral strains and reduce significantly, if not totally eliminate, the need for costly post-vaccination surveillance.  The ease of administration of this vaccine (eye drop or one single injection) would further contribute to its relevance in the field.  These characteristics are considered essential for the product profile of an AI vaccine that can contribute in a meaningful way to the livelihoods of poultry smallholders.

## Introduction

Although more than 30 epizootics of high pathogenicity Avian Influenza (HPAI) have been reported in poultry ( *Gallus domesticus*) and other birds since 1959, vaccination of Poultry has only been added as a control tool since 1995 ( Swayne *et al*., 2014). In recent outbreaks, vaccination has been used only in about 19% of the countries experiencing HPAI 15 countries out of 80 countries), showing that vaccination is not the most common and immediate response to an outbreak ( Swayne *et al*., 2011). The nature of the AI virus, with the rapid emergence of new field viral strains, through genetic drifts of HPAI viruses, has affected the effectiveness of vaccines. Vaccination along with other measures has been successful in eradicating AI in very few countries where the competence of national veterinary services (e.g. France, the Netherlands) or the geography and bird density (Hong Kong) have favorably contributed to the outcome. In the four countries (China, Egypt, Indonesia and Vietnam) where massive vaccination campaigns have been initiated since 2004 (accounting for more than 99% of the use of AI vaccines), HPAI virus is now endemic, outlining the difficulty to eradicate AI in countries with a high percentage of backyard poultry ( Swayne *et al*., 2011). A number of factors are limiting the effectiveness of AI vaccination in smallholder settings and are discussed in the following sections along with the characteristics of the ideal vaccine to target the poultry smallholder segment.

## AI – etiology and epidemiology

Influenza viruses belong to the Orthomyxoviridae family, causing respiratory disease of the upper respiratory tract in humans, avian species, and a variety of mammal species. Orthomyxoviruses are classified as Types A, B or C with Avian influenza caused by a highly mutable Type A influenza virus.

The replication of this single stranded RNA virus is highly variable, resulting in a constantly evolving and highly mutable virus ( Swayne *et al*., 2013). The lipid envelope of the virus makes it unstable and relatively susceptible to environmental destruction from ultraviolet light, chemicals, or desiccation. The hemagglutinin (HA or H) antigen is a protein providing the mechanism for the entry into the host cell while the neuraminidase (NA or N) protein allows the exit of newly replicated virions from the host cells. The HA protein is the major antigen stimulating the host immune response with protective antibodies against clinical signs and mortality. There are at least 16 H strains and 9 N strains, which can result in 144 possible HN combinations. This forms the basis of the serological classification using the hemagglutinin inhibition and neuraminidase inhibition tests. The nomenclature of the virus is based on the HN subtype, influenza type, host species, sample location, strain number and year of isolation, e.g. H5N1 A/goose/Guangdong/1/1996.

The antigenic variation of the HA and NA surface glycoproteins occurs at a high frequency through minor “drift” changes and may be associated with the immune pressure exerted by the vaccination of birds. Major antigenic “shift” in the HA and NA coding proteins, the result of genetic re-assortment between gene segments of two different influenza virus strains (subtypes) in host cells, commonly occurs especially when domestic waterfowl and poultry are in close proximity as often seen in developing countries under smallholder settings. Ducks are often silent carriers of the AI virus, constituting an additional challenge for the control of AI ( Swayne *et al*., 2013). AI viruses in poultry are classified as either low pathogenic (LPAI) or highly pathogenic (HPAI), based on the clinical signs and mortality, using the definition established by the World Organization for Animal Health ( OIE, 2018). During the period January 2013 to February 2019, HPAI in domestic poultry was reported in 68 countries and territories with a total of 7,270 outbreaks ( OIE, 2019). In most of the countries in Asia and Africa, the poultry sector is dominated by smallholder farmers.

During the same period, a total of 12 subtypes of AI viruses were reported ( OIE, 2019), with subtypes H5N1, H5N2 and H5N8 widespread and more frequently reported ( Table 1).

**Table 1.** Distribution of different HPAI subtypes in domestic birds, by region (January 2013 – February 2019).

Region	HPAI subtypes
Africa	H5N1, H5N2, H5N8
Americas	H5N1, H5N2, H5N8, H7N3, H7N8, H7N9
Asia	H5N1, H5N2, H5N3, H5N6, H5N8, H7N9
Europe	H5N1, H5N2, H5N5, H5N6, H5N8, H5N9, H7N7
Oceania	H7N2

LPAI outbreaks with viruses of H5 and H7 subtypes are also reportable to the OIE as there is a risk of the viruses becoming highly pathogenic by mutation. During the period January 2013–February 2019, the vast majority of these outbreaks were reported in Asia, Europe, the Americas and a few in Africa (mainly in South Africa).

H5N1 HPAI viruses have been sub-classified according to Clades, or groups of AI viruses that share a common ancestor ( WHO, 2011). There are at least 10 Clade groups currently identified by the OIE and FAO network of expertise on animal influenza (OFFLU). Both antigenic shift and drift are important mechanisms for the evolution of the virus. The presence of co-circulating subtypes among dense populations of birds adds extra pressure for antigenic shift. Intrinsic subtype specific antigenic drift is associated with the frequency and distribution of infection in a poultry population as naive populations become exposed to new variants. Vaccination is also believed to exert a selection pressure on the virus by increasing the mutation rate by several orders of magnitude ( Swayne *et al*., 2014). In addition, the evolution of new clade types can change the morbidity and mortality. Prior to 2012, clade 2.2 (Indonesia) and clade 1.1 (Cambodia) were predominant, with mortality in domestic ducks of less than 10%. Following the introduction of clade 3.2.1, the mortality reported in ducks is greater than 40% and up to 90% depending on the age of the ducks. As a virus is transferred from wild birds to poultry, natural selection within the host favours greatly adapted strains, which often become more pathogenic for both LPAI and HPAI pathotypes ( Swayne *et al*., 2013).

The type of poultry production is also critical to assess the risk of exposure. Birds kept for longer periods (laying hens, breeder birds and slow-growth meat birds) have a longer duration of potential exposure to AI virus than short-lived poultry (broiler-type meat birds). The concurrent presence of immunosuppressive agents and conditions for example, reduce the infectious dose and has been associated with morbidity and mortality due to both LPAI and HPAI infections. Therefore, general health screening for concurrent diseases in poultry, often difficult to achieve under smallholder settings, is important during immunization and for the overall control of avian influenza. 

AI outbreaks during the period January 2013–February 2019 resulted in the loss of approximately 128 million birds, with more than half (57.6%) of the reported losses in Asia, followed by the Americas (22.1%) and Europe (13.4%) ( OIE, 2019). Losses in Africa accounted for only 6.5% of the total losses ( Table 2).

**Table 2.** Distribution of mortality in domestic birds, by region (January 2013 – February 2019).

Region	Losses	Percentage of total
Africa	8,339,384	6.5%
Americas	28,224,324	22.1%
Asia	73,673,631	57.6%
Europe	17,187,364	13.4%
Oceania	490,000	0.4%
Total	127,914,703	100 %

## AI vaccines and vaccination

### AI vaccine types and production

Poultry AI vaccines used in the field are based on five technologies: 1) wild-type or reverse genetics whole AI virus grown in embryonated chicken eggs, then chemically inactivated and adjuvanted; 2) HA antigen or virus-like particles produced in insect cells by a genetically engineered baculovirus; 3) HA DNA vaccine adjuvanted; 4) recombinant technologies utilizing live virus vectors to express AI virus HA and in some cases NA gene inserts (recombinant Herpes turkey virus (rHVT-AIV), recombinant Newcastle disease virus (rNDV-AIV) and recombinant Fowl pox virus (rFPV-AIV)) and, 5) defective-replicating alphavirus (defective Venezuelan Equine Encephalitis virus with H5 AI virus gene insert (D. E. Swayne, personal communication). 

The recombinant ND-vectored vaccines used for the control of AI are broadly divided into two categories ( Suarez *et al*., 2017). One alternative uses reverse genetic technology to insert an AI gene sequence coding for a specific protein into the ND virus thus producing a recombinant ND virus expressing that protein. After viral multiplication in embryonated chicken eggs, the recombinant viruses recovered are inactivated and adjuvanted for the production of the AI vaccine. This technology was recently patented in the USA by Laboratorio Avimex ( Lozano-Dubernard *et al*., 2015) and presents the advantage of being safe to use in BSL-2 production facilities, more readily available in developing countries.

The second alternative applies the reverse technology to produce live ND vector vaccine viruses, often using the more aggressive LaSota vaccinal strain as the backbone, which express HA genes (H5, H6, H7 and H9 inserts). One of the primary benefits of this alternative is that the live virus replicates on mucosal surfaces, can be administered by mass application such as water or aerosol application, thus reducing the overall cost of vaccination and may be administered to other species of poultry, especially ducks. Conversely, one of the main concerns is related to the level of maternal ND antibodies in chicks which may interfere with the replication of the recombinant ND viruses and overall efficacy of the vaccine. In addition to being considered as genetically modified organisms, the potential of live ND vectored vaccines to spread to non-target species and to unvaccinated flocks often raises concerns during the regulatory review and vaccine licensing process ( Suarez *et al*., 2017).

There are several new experimental AI vaccine approaches not currently licensed for commercial use including wild type or attenuated LPAI; use of various vectors e.g. adenovirus, salmonella, avian leukosis and vaccinia; eukaryotic systems e.g. plants; and DNA vaccines, with a main objective being a universal vaccine covering against all subtypes of HPAI. Of course, this would be an ideal scenario. However current opinion is that there is little expectation that such vaccine will be developed to licensure in the foreseeable future.

### Multivalent vaccines commercially available

Review of the vaccine database from The Center for Food Security and Public Health, Iowa State University showed that of 43 manufacturers listing vaccines for the control of Avian Influenza, 31 (72%) are currently manufacturing vaccines combining AI with other antigens ( CFSPH, 2018). These manufacturers are based in 11 different countries on five continents but the majority of them (71%) are located in Asia (22 of the 31) where AI vaccination has been practiced since 1995. Manufacturers located in Europe and North America are producing vaccines mostly for export and for the setting-up of emergency stocks. There is currently just one manufacturer of AI vaccines on the African continent, although it is believed that others are at a planning stage.

 The combination vaccines produced are either AI + ND only (26 manufacturers, 84%), AI +ND + other antigens (15 manufacturers, 48%) and AI + another antigen but not ND (3 manufacturers, 10%), these numbers accounting for the fact that some manufacturers are producing more than one type of AI combination vaccine. 

### Use of AI combination vaccines by the poultry sector

Based on the list of antigens combined with AI presented previously, it is evident that these vaccines are mostly used in the egg laying and breeder poultry sectors and not by poultry smallholders. As might be expected from this wide range of vaccines and the disease challenges experienced in the field, there are a multitude of vaccination schedules used by poultry producers. 

As an example, a recent study in Indonesia by Tarigan *et al*. (2018) showed that there were broadly three types of schedule for the administration of AI vaccines to commercial laying birds:

-   two or three vaccinations before 19 weeks of age (start of laying period)

-   two vaccinations before 19 weeks of age and one vaccination after 19 weeks

-   three or four vaccinations before 19 weeks and 2 or 3 vaccinations after 19 weeks

Irrespective of the vaccination schedule or the specific vaccines used, Tarigan *et al*. (2018) demonstrated that the birds were not protected throughout production. Without vaccination after 19 weeks, the birds failed to be protected after 38 weeks of age; with vaccination after 19 weeks, the birds failed to be protected around 58 weeks of age. The data clearly demonstrated showed that with laying or breeder birds the duration of immunity is one of the main limitation issues for life cycle protection in the field, especially in the smallholder setting.

Among the recombinant viral-vectored vaccines, the rHVT-AIV has the advantage that it can be used in day-old chicks at the hatchery. This vaccine has shown some good results in field studies in Egypt and is currently licensed in five countries (Bangladesh, Egypt, Mexico, Vietnam and the USA ( von Dubschuetz, 2013).

### Need for regular AI vaccine reformulation

Due to the antigenic drift of the AI virus in the field, there is a need to constantly update the composition of AI vaccines. This can be achieved only through constant monitoring of the virus strains in the field, rapid regulatory review and approval process and, good manufacturing processes. As the largest user of AI vaccines, the evolution over time of the vaccinal strains used in China is quite indicative of the need for regular reformulation, both for killed and live vaccines ( Table 3).

**Table 3.** Evolution of the H5N1 viral strains used in killed vaccines in China (2004–2014).

Year	Viral strains in H5N1 vaccines used in China
2004	Heterologous H5N2 strain, killed
2005	H5N1 Re-1, killed
2006 – 2012	Killed H5N1 Re-4, Re-5, Re-6 and bivalent vaccines killed H5N1 Re-1/Re-4, Re-4/Re-5 and Re-4/Re-6
2014	H5N1 Re-7, killed

Over the course of 10 years, the control of H5N1 in China has required the introduction of seven different killed vaccines. In addition, H5N1 has also been combined with H9N2 for better vaccination coverage, adding two new killed vaccine formulations ( Fan *et al*., 2015). In 2017, a vaccine combining H7N9 with H5N1 was approved for use in poultry ( Shi *et al*., 2018). The need for the regular updating of H5N1 viral subtypes is also required for live recombinant NDV-vectored vaccines, with three different formulations (rLH5-1, rLH5-5 and rLH5-6) approved since 2005 ( Fan *et al*., 2015). The need for regular updating of vaccinal strains was also reported by Tarigan *et al*. (2018) in Indonesia, another country using vaccination to control avian influenza on a wide scale since the first outbreak was reported in 2003 ( Table 4).

**Table 4.** Evolution of the H5N1 viral strains used in killed vaccines in Indonesia (2004–2012).

Year	Viral strains in H5N1 vaccines used in Indonesia
2004	H5N1 subclade 2.1.1
2008	H5N1 subclade 2.1.2 and 2.1.3
2012	H5N1 subclade 2.3.2.1

Due to the antigenic drift of the AI virus in the field, the geographical coverage of AI vaccines is very specific and sometimes limited to certain provinces or districts in a country. Therefore, most AI vaccines administered in China, Pakistan, Indonesia and Egypt are produced by local manufacturers.

### Key learnings from national AI vaccination campaigns

Since 2002 with the use of vaccines to control H5 HPAI in Hong Kong, mass AI vaccination campaigns have been implemented in China, Vietnam, Indonesia, Egypt and Mexico. Key learnings were shared by these countries during the OFFLU technical meeting in Beijing, China and reported in the recommendations issued ( OFFLU, 2013). A number of authors have also reviewed the performance of vaccination campaigns in Egypt ( Kaoud, 2017), Indonesia ( Swayne *et al*., 2015), Asia ( Peyre *et al*., 2009) and globally ( Pavade *et al*., 2011) as well as the economic cost and benefits ( Hinrichs & Otte, 2012; Hinrichs, 2013; Sun *et al*., 2017). 

The key learnings from these national AI vaccination campaigns are summarized below:

•   Antigenic drift occurs with all AI viruses and can reduce the effectiveness of vaccination over time. It is essential to use vaccinal strains with sufficient quantities of antigens reasonably well matched with circulating strains of AI virus.

•   Vaccination with high-quality, registered vaccines, according to established protocols reduces resistance to infection, decreases viral shedding and decreases the probability of infection to poultry, other animals and humans.

•   Vaccination only partially reduces viral shedding and can promote, especially if not conducted correctly, depending on the choice of vaccine, mode and frequency of administration, the selection of mutation in the circulating virus.

•   Vaccination alone cannot eliminate the virus and is only meant to be part of an integrated, holistic control program adapted to local conditions

•   It is difficult to maintain high level flock immunity in some poultry populations, especially at small production and backyard levels.

•   Vaccination is logistically demanding, and additional costs will be initially incurred in countries lacking efficient cold chain distribution networks required for most vaccines.

•   Vaccination is expensive due to the need to conduct high quality post-vaccination surveillance to monitor the genetic and antigenic characteristics of circulating field viruses.

•   The return on investment of vaccination must be carefully considered before adding vaccination as part of an AI control programme.

In Indonesia, a survey of commercial egg producers ( Brum, 2013) showed that AI vaccinations were ineffective for the following reasons: poor selection of vaccinal strains vs. field challenge, first AI vaccine given too late and, not enough booster vaccinations, especially during the period of egg production (when vaccination will cause a reduction in egg production). Farmers also lacked independent high-quality technical support and 55% of the respondents said that they needed better information on vaccine selection and the vaccination schedule.

Regarding the situation in countries with large backyard poultry production, Alders *et al*. (2007); Gardner & Alders (2014); Pavade *et al*. (2011) and Sims *et al*. (2016) have provided some additional points to consider:

•   Backyard poultry are extremely hard to reach, and the exercise remains extremely costly. Thus, vaccination should not be started if adequate funding for the preparation, implementation and monitoring cannot be guaranteed.

•   As a rule, it is not recommended to vaccinate backyard poultry since maintaining adequate levels of immunity is extremely difficult.

•   Community vaccinators are often reluctant to vaccinate backyard birds under 2 months of age for fear of killing them, thereby missing about 50% of the birds at risk.

•   Vaccination is not a substitute for other important measures like biosecurity and can give farmers a false sense of security.

•   Poor vaccination practices, insufficient vaccination coverage and lack of post-vaccination monitoring (as often seen in developing countries with large backyard poultry populations) can contribute to an endemic situation.

## Overview on government AI control

Information on national regulations and policies related to AI vaccination is available via the World Animal Health Information System (WAHIS) interface hosted by the OIE ( OIE, 2019). This database is built from information submitted to the OIE by its 184 country members. Among the parameters recorded, the following information is relevant to this paper: vaccination prohibited and official vaccination. It must be noted that official vaccination is not necessarily the converse of vaccination prohibited. Vaccination against poultry diseases may be the result of official vaccination and / or voluntary vaccination.

According to the OIE website ( OIE, 2019), the countries with official AI vaccination programs are Mexico, Egypt, Pakistan, Kazakhstan and Russia. China also has official AI vaccination. Vaccination is also allowed in Bangladesh where import permits are delivered to the private sector and killed AI vaccines imported (no combination vaccines used). Vietnam, Egypt and Indonesia have transitioned from mass vaccination to targeted vaccination ( OFFLU, 2013). The cost of AI vaccination is often shared between governments and the private sector. For instance, in Vietnam, farmers with >2,000 birds pay the full cost of vaccination while farmers with <2,000 birds receive some provincial subsidies. 

Regional organizations like the African Union and the Association of South-East Asian Nations do not have specific policies regarding AI except for the desire to strengthen the linkages within countries and across borders, for the sharing of information and knowledge and for the development of partnership between all stakeholders in public and private sectors and civil society.

At the intergovernmental level, the Network of Expertise on Animal Influenza (OFFLU), a joint OIE-FAO technical committee with a worldwide network of contributors, issued in 2013 a series of recommendations on vaccination and vaccines ( OFFLU, 2013).

## The ideal AI vaccine candidate for poultry smallholders

The desired attributes (product profile) for AI vaccines targeting the smallholder segment are presented in Table 5.

**Table 5.** Desired attributes of AI vaccines addressing the smallholder segment.

Desired attribute	Current situation
Inexpensive	Current cost for inactivated AIV vaccine: $0.03–0.10/dose plus cost of administration ($0.05–0.07 per dose for individual handling and injection)
Use in multiple avian species	Most vaccines are used in meat, layer and breeder chickens although a large number of doses also used in ducks; minor amounts in turkeys, geese, quail, etc.
Single dose protection	Most situations require a minimum of 2 doses; prime-boost scenario is optimal with revaccination in long-lived birds at 6–12-month intervals
Mass application	95.5% is inactivated vaccine administered by handling and injecting individual birds and 4.5% as vectored vaccine given by mass spray vaccination
Identify infected birds in vaccinated population (DIVA)	Serological differentiation tests are available, but only minor use. Most vaccine applied without using a serological DIVA strategy for surveillance
Overcome maternal antibody interference	Maternal antibody to AIV hemagglutinin or virus vector inhibits primary immune response. Initial vaccination must be timed for declining maternal antibody titers to allow optimal primary immune response
Given at 1 day of age in hatchery or *in ovo*	Inactivated vaccine provides poor protection when given at 1 day of age. Vectored vaccines can be given at 1 day of age, but generally require a boost with inactivated vaccine 10 days or more later
Universal vaccine	The majority of inactivated whole AIV vaccines use reverse genetic generated vaccine seed strains to antigenically match field viruses. The vaccinal strain of virus should also be a strong immunogen
Thermostable	Killed AI vaccines, rNDV-AI and rFPV-AI vaccines require refrigeration and rHVT-AI vaccine must be stored in liquid nitrogen

Based on the views of a number of experts from academia, governments, intergovernmental organizations and the private sector, the top desired attributes for the smallholder segment are listed in Table 6.

**Table 6.** Desired attributes of AI vaccines addressing the smallholder segment.

Desired attribute	Rationale
Universal vaccine	The antigenic drift of the AI viruses in the field requires a constant surveillance usually poorly performed by national veterinary services in developing countries due to the lack of proper facilities, human and financial resources. In addition, the availability of universal vaccines would eliminate the need to regularly update vaccines, often affected by inefficient national regulatory review and approval processes for vaccines
Use in multiple species	Ducks often act as silent carriers, shedding AI virus without expressing clinical signs. An effective vaccine in ducks as well as chickens would be of greater value, especially in South East Asia
Single dose protection	The biggest logistical hurdle and highest cost of AI vaccination is related to the handling and injection of each bird. A vaccine conferring at least a 6-month protection after one single administration would maximize the value of mass vaccination campaigns

Due to the genetic drift of the AI viruses, the current commercial vaccines have a limited geographical coverage and use. The development of a universal vaccine would allow for the same commercial vaccine to be used in all major countries currently vaccinating against AI (China, Egypt, Indonesia, Vietnam and Mexico). Such a vaccine would be also available in countries considering adding vaccination to their AI control measures even though the current trend shows that fewer countries are willing to initiate AI vaccination. 

## Conclusion

Vaccination against HPAI has been used as one of the tools in national AI control programmes with varying degrees of successes. Few countries have been able to eradicate HPAI and these countries have typically relied on extremely competent national veterinary services and / or had limited poultry population at risk. Current vaccines, when well-matched to field strains (in quantity and quality), administered properly (timing, frequency and method of administration) and combined with a wide range of other measures have prevented financial losses in poultry farms and reduced the risks to human populations.

The cost of AI vaccines is only a small part of the overall cost of vaccinating poultry against AI, especially when targeting poultry smallholders in lower- and middle-income countries. The current vaccine production technologies and vaccines have not been able to address the cost inherent to their use. In order to represent a significant advance, the profile for vaccine candidates must include protection against all viral strains (universal vaccine), efficacy in ducks with reduction in viral shedding and, protection after a single administration. Without the inclusion of these properties in the vaccine profile, it is unlikely that any vaccine candidate would add significant value to the current AI vaccine market and thus warrant the investment of funds for its development.

## Data availability

No data are associated with this article.

## Disclaimer

The views expressed in this article are those of the authors. Publication in Gates Open Research does not imply endorsement by the Gates Foundation.
