# Supplementary material for: Are current avian influenza vaccines a solution for smallholder poultry farmers?
Source: Gates Open Res. 2020 Aug 26;4:122. [Version 1] doi: 10.12688/gatesopenres.13171.1 (PMC7578560; doi:10.12688/gatesopenres.13171.1)
Supplement: Peer review history

# Peer review: Reviewer response for version 1

**DOI:** 10.21956/gatesopenres.14370.r29532

**Published:** 2020-10-26

**Authors:** Leslie D. Sims

**Affiliations:** 1 Asia Pacific Veterinary Information Services Pty. Ltd, Montmorency, Vic, Australia

- The paper provides an interesting commentary and suggestions on vaccination against highly pathogenic avian influenza. In general, the recommendations are reasonable that better vaccines with the properties described would be valuable. However, there are areas where revision should be considered given the population of farmers targeted has not been fully defined, all reasons for vaccination have not been covered, the authors have not always provided sufficient balance between the problems associated with vaccination and the solutions available, and could have provided more information on new developments in vaccination. In addition, availability of suitable vaccines is not necessarily the main reason for not applying vaccination.  
- The paper focuses on mass public sector vaccination programmes, in particular vaccination against Gs/GD/96-lineage highly pathogenic H5Nx avian influenza. However, in several countries where these viruses are endemic and vaccination is used against this disease, no formal government programme is in place (the role of government is to approve vaccines for use). It is up to individual farmers to purchase and administer the vaccine (or get someone to do it for them).  
- As the authors point out, vaccination of small flocks against avian influenza can be difficult due to logistical difficulties, and rapid turnover in multi-age flocks. It has been demonstrated in Egypt and Indonesia that it is not sustainable as a government-sponsored programme. However, there is no technical reason why a well-matched, relatively inexpensive, killed antigen vaccine cannot be administered to birds owned by small scale producers and interested in protecting their flock provided there is a suitable veterinary professional or paraprofessional to perform the work and the owner is willing to pay for vaccination. This is being done already or is potentially available in both Vietnam and China – two countries that first introduced mass vaccination against Gs/GD/96-lineage H5Nx viruses. Issues that arise are those of application, including availability of a well-matched vaccine, providing sufficient doses at an appropriate time, a lack of interest/demand for vaccination from some very small scale, low input producers. Field observations and reports on mass vaccination in places with large numbers of smaller scale producers do suggest it is not always done well, based on levels of antibodies reported in places where these have been measured post-vaccination. Nevertheless, a community-based veterinary paraprofessional could provide this service to any household in much of SE Asia if the demand was there and vaccination was allowed (which it isn’t in a number of countries where Gs/GD/96-lineage H5Nx viruses are endemic or where cases recur).  
- The term “small scale producers” is not defined. If this refers to household flocks of, say, 10 to 30 birds reared extensively then there are many logistical problems in delivering vaccine to these flocks. Many are multi-age, owners are not interested in vaccination and delivering vaccine to all birds at the appropriate age is difficult. One problem is that vaccine is produced for larger flocks in multidose bottles (see recommendations from OFFU meeting in Beijing). Another part of the problem is that the amount paid for administering vaccination in some places is too low to justify the expense of travel by veterinary paraprofessionals. However, in places where highly pathogenic avian influenza occurs seasonally and regularly, it would be possible to provide vaccination coverage to flocks in an area if required/demanded.  
- In places where outbreaks occur sporadically the signals and need for vaccination are weak.
- Much of the infection with Gs/GD/96-lineage virus occurs in domestic ducks and for many strains the level of mortality produced is relatively low and age-dependent. There is little incentive for duck farmers to vaccinate unless they have experienced losses from this disease previously or have experienced losses due to government action when an outbreak occurs on nearby farms (such as whole village culls). 
- Even if a universal HPAI vaccine for poultry could be developed there is no guarantee it would be used by all producers, especially those rearing poultry in low input systems or for short-lived birds, especially if there is an outlet for birds in the event of an outbreak. Many farmers will sell birds when they see early signs of mortality and those with very small flocks are less likely to vaccinate than those with larger flocks (Delabouglise *et al.*, 2020 ^1^). In low input systems the loss of some birds is expected.
- Vaccination is a cost to producers and in many countries rearing of broilers is a low margin business. This will result in some farmers choosing not to vaccinate even if effective vaccines are available.
- The paper highlights the potential downsides of vaccination without necessarily balancing this with information on methods to overcome the concerns. For example, much is made of the antigenic variation restricting the effective life of vaccines. This has been the case in several countries in which the virus remains poorly controlled but not in others. Action has been taken to ensure appropriate vaccines are available in most countries albeit more slowly than required.
  Several vaccines already offer broad cross protection against different clades within the Gs/GD/96-lineage.
- The paper does not cover one of the biggest constraints to vaccination which is the right for farmers to access vaccines. In a number of countries, H5 avian influenza vaccine cannot be used legally despite the virus being endemic.
- There is also no mention of ring vaccination, as used in some countries (but discouraged in others) around outbreaks. There is also no discussion on the benefits of having immune birds delivered to live bird markets as has been seen with both H5Nx and H7N9 viruses reducing the likelihood of infection in birds and spill over to humans.
- The following points on the authors text (in italics) should be considered if/when revising the paper. Note that some of the references cited were published after this paper was submitted but should still be considered. Papers cited on advances in vaccines are only examples. 

**Title: **

- The article is discussing vaccination against highly pathogenic avian influenza so this should be included in the tile.

**Extracts from the abstract :**

*Vaccination against highly pathogenic avian influenza (HPAI) viruses, along with other measures, was successful in eradicating AI in very few countries where the competence of national veterinary services or the geography and bird density have contributed favorably to the outcome.  *

- The objective of vaccination against highly pathogenic avian influenza in most places was not elimination but, rather, to reduce levels of infection and the threat to humans. This decision was made because it was not possible to eliminate the virus using the methods generally applied, based on stamping out and movement management. Vaccination was used in Hong Kong and Mexico to help eliminate particular strains of highly pathogenic H5 avian influenza virus and in Hong Kong to supplement measures in place to prevent infection in markets.  
- Eradicate vs eliminate? WHO uses the former for global “elimination”.  I prefer use of elimination.

*The main constraints to an effective AI vaccination are vaccine composition matching field strains, reliable cold chain, and logistics to target all poultry smallholders, constraints related to the availability of sufficient financial and human resources.  *

- Matching vaccines to field strains is not as important as proposed here in all places where vaccine is being used (it is an issue that can be overcome, and methods are available for rapid production of matched vaccines). In southern Vietnam, the same vaccine remained suitable from 2005 for at least 5 years against clade 1 viruses until the dominant strain changed due to introduction of a new clade. Cold chains are important but not a constraint for delivering other poultry vaccines that require similar storage in SE Asia. Vaccination does not need to target all small-scale producers if the goal is disease containment. The cost of vaccination is only one of the issues. Other important constraints to use of vaccine are the life span of birds (e.g. very short life span for broilers in places such as Indonesia), interference from maternal antibody and as mentioned above not having the right to use vaccines due to national bans (e.g. not currently permitted in a number of Asian countries even in countries where the virus is endemic).

*When not conducted properly, vaccination can also contribute to the emergence of new field viral strains, through genetic drifts of HPAI viruses.  *

- Note that antigenic variation also occurs when vaccination is not used – e.g. H6 in China (Wang *et al.,* 2014 ^2^). Antigenic change will occur slowly in places where vaccinated or immune birds are rarely exposed to virus. In Vietnam and Indonesia, the bigger driver of vaccination failure was the introduction of a novel strain of virus from outside the country. Antigenic drift was not a factor for Clade 1 viruses and vaccines in southern Vietnam over a number of years. Circulation of multiple strains of virus is also a constraint – matching becomes more difficult when more than one antigenic variant is circulating.
- Would use of vaccine be regarded as improper in places where infection is endemic and high-level population immunity across areas is hard to achieve but good immunity at the individual farm level is achievable? Much depends on the purpose of the vaccination programme.

*While new technologies have improved the possibility to produce high quality vaccines matching field strains, recurrent issues like post-vaccination field surveillance and vaccination coverage continue to limit the relevance of AI vaccination in smallholder settings. *

- The extent of post-vaccination surveillance depends on the purpose of vaccination.  In a country where the virus is endemic and there is no possibility of elimination in the short to medium terms (2 to 5 years or longer), expensive surveillance systems for all flocks cannot be justified.

*A “gamechanger” vaccine targeting smallholders should be universal to protect against all field viral strains and reduce significantly, if not totally eliminate, the need for costly post-vaccination surveillance. The ease of administration of this vaccine (eye drop or one single injection) would further contribute to its relevance in the field.  These characteristics are considered essential for the product profile of an AI vaccine that can contribute in a meaningful way to the livelihoods of poultry smallholders.*

- It would be good to have such a vaccine, but we can still vaccinate smallholder flocks even with existing products, provided care is taken to use the most appropriate vaccine. The main post-vaccination surveillance required is detection of antigenic variant strains. This is not expensive. Surveillance is only costly if the goal is virus elimination and detection of all infected flocks. This is not the case in places where viruses remain endemic. Surveillance programmes recommended for use in Europe are expensive and can be a barrier for use of vaccines.

**Main text:**

*Although more than 30 epizootics of high pathogenicity Avian Influenza (HPAI) have been reported in poultry (Gallus domesticus) and other birds since 1959, vaccination of poultry has only been added as a control tool since 1995 (Swayne et al., 2014). In recent outbreaks, vaccination has been used only in about 19% of the countries experiencing HPAI 15 countries out of 80 countries), showing that vaccination is not the most common and immediate response to an outbreak (Swayne et al., 2011). *

- Vaccination is not (in most situations) the most common response. However, the reason for this low uptake is that in high income countries, if virus is detected early, stamping out is used successfully to eliminate the virus.  There are some exceptions to this rule. Trade restrictions can also inhibit use of vaccines. There are also situations where vaccination could be used as an early response to an outbreak, but trade issues inhibit its use.   

*The nature of the AI virus, with the rapid emergence of new field viral strains, through genetic drifts of HPAI viruses, has affected the effectiveness of vaccines.*

- In some countries there has been rapid emergence of antigenic variants within a few years, in other countries the vaccine strains used have been suitable for multiple years. However, most countries that are using vaccine have systems in place to update vaccine antigens.

*Vaccination along with other measures has been successful in eradicating AI in very few countries where the competence of national veterinary services (e.g. France, the Netherlands) or the geography and bird density (Hong Kong) have favorably contributed to the outcome.*

- In which situation has vaccination been used to eliminate avian influenza in France or the Netherlands? They both used vaccination selectively as a preventive measure in 2006 but this was abandoned, in part, presumably because of the cost of the required surveillance. Hong Kong SAR farms were densely packed, and vaccination was used as a measure to help prevent infection not only in farms but downstream in live poultry markets. Vaccination was added to the measures to contain the outbreak in 2002 and 2003 but became a compulsory preventive measure in December 2003 for any bird (imported from mainland China or locally reared) destined to be sold in a live poultry market in Hong Kong SAR. No cases occurred in Hong Kong until 2008 when an antigenic variant strain affected one poultry farm and was detected in several markets. No cases have been recorded since. It was the structure of the industry in Hong Kong SAR that resulted in routine preventive vaccination (live birds sales, import of live birds from mainland China and biosecurity systems on farms that could not be improved sufficiently to be confident of preventing all viral incursions).

*In the four countries (China, Egypt, Indonesia and Vietnam) where massive vaccination campaigns have been initiated since 2004 (accounting for more than 99% of the use of AI vaccines), HPAI virus is now endemic, outlining the difficulty to eradicate AI in countries with a high percentage of backyard poultry (Swayne et al., 2011). *

- The virus was endemic before vaccination commenced in these countries and was used because virus elimination was not possible owing to the nature of the poultry production and marketing sector (Sims, 2016). Vaccination did not result in endemic infection – it was a response to it.  Note also that certain strains of virus have disappeared (e.g. clade 7 viruses in China). Note also that vaccination has been used in Bangladesh for a number of years now. Vaccination has also been used illegally in a number of countries where it is banned. The presence of a high proportion of smallholder flocks is not necessarily an impediment to elimination (e.g. Thailand) even when vaccination is not used. Note that there is considerably more usage of H9N2 vaccination across Eurasia than against H5Nx so when referring to AI vaccines ensure this states it is for HP viruses.

*Orthomyxoviruses are classified as Types A, B or C with Avian influenza caused by a highly mutable Type A influenza virus.*

- And Type D. All type A viruses are highly mutable, not just avian influenza viruses

*The antigenic variation of the HA and NA surface glycoproteins occurs at a high frequency through minor “drift” changes and may be associated with the immune pressure exerted by the vaccination of birds. *

- But there are other drivers apart from vaccination (see Wong *et al.,* re H6). In some cases, vaccination has probably resulted in selection of an existing antigenic variant (e.g. H9N2 in Korea).

*Major antigenic “shift” in the HA and NA coding proteins, the result of genetic re-assortment between gene segments of two different influenza virus strains (subtypes) in host cells, commonly occurs especially when* *domestic waterfowl and poultry are in close proximity as often seen in developing countries under smallholder settings. *

- Does this reassortment occur in smallholder settings or in live bird markets? Also much of the recent reassortment has probably occurred in wild birds (King et al., 2020 ^3^). There is a much greater likelihood of reassortment in environments where multiple strains of virus are circulating as was the case in markets in Hong Kong in 1997. Most of this reassortment involves internal protein genes. The H5N1 combination was favoured until the emergence of clade 2.3.4.4 viruses which have been shown to readily acquire different NA genes. 
- For Gs/GD/96-lineage viruses the HP H5 HA has not been replaced since 1996 so there has not been a true shift (involving a change in subtype). Drift has occurred and certain HA clades have emerged that are so antigenically dissimilar that vaccines are ineffective when a novel strain is introduced to a new area. For example, Indonesia with the introduction of Clade 2.3.2.1 viruses. 

*Ducks are often silent carriers of the AI virus, constituting an additional challenge for the control of AI (Swayne et al., 2013). *

- It is preferable to state that ducks can be infected silently given individual ducks rarely shed viable virus for a period beyond 10 days. They are not true carriers.

*AI viruses in poultry are classified as either low pathogenic (LPAI) or highly pathogenic (HPAI), based on the clinical signs and mortality, using the definition established by the World Organization for Animal Health (OIE, 2018). During the period January 2013 to February 2019, HPAI in domestic poultry was reported in 68 countries and territories with a total of 7,270 outbreaks (OIE, 2019). *

- The number of outbreaks is in some ways misleading given most cases of infection are not reported/detected in countries where the virus is endemic. The majority of these HPAI outbreaks were caused by Gs/GD-96-lineage H5Nx viruses.

*In most of the countries in Asia and Africa, the poultry sector is dominated by smallholder farmers.*

- It would be good to define what you mean by smallholder farmers. It is much more difficult (but not impossible) to implement vaccination for small multi-age flocks of say <50 head than a commercial flock of say 500 birds.  Note in Africa there is no vaccination against HPAI (officially), except in Egypt.  

*H5N1 HPAI viruses have been sub-classified according to Clades, or groups of AI viruses that share a common ancestor (WHO, 2011). There are at least 10 Clade groups currently identified by the OIE and FAO network of expertise on animal influenza (OFFLU). *

- This system applies to Gs/GD-96-lineage H5Nx viruses, not other H5 HPAI viruses. Although the initial classification divided the viruses into 10 clades 0 to 9, most of these clades have extinguished but viruses within Clade 2 have continued to evolve to form a number of 5 ^th^ order clades, especially within clades 2.3.2.1 and 2.3.4.4

*Both antigenic shift and drift are important mechanisms for the evolution of the virus. The presence of co-circulating subtypes among dense populations of birds adds extra pressure for antigenic shift.*

- Delete – mentioned already but see earlier comments about shift and reassortment.

*Vaccination is also believed to exert a selection pressure on the virus by increasing the mutation rate by several orders of magnitude (Swayne et al., 2014). *

- This is probably true in places where there is a high likelihood of infection in vaccinated flocks (e.g. Egypt) but not in situations where there is a good match between circulating virus and the vaccine antigen or where incursions of virus are infrequent. Experimentally it is possible to select for antigenic variants using antiserum suggesting a role for antibodies, but we still do not know the precise contribution of vaccination vs natural infection, especially in ducks.

*In addition, the evolution of new clade types can change the morbidity and mortality. Prior to 2012, clade 2.2 (Indonesia) and clade 1.1 (Cambodia) were predominant, with mortality in domestic ducks of less than 10%. Following the introduction of clade 3.2.1….*

- Indonesia was clade 2.1 and derivatives. The introduced virus was clade 2.3.2.1c to both. Note that many healthy market-weight ducks have been found infected with clade 2.3.2.1c viruses based on results of market surveillance in Cambodia so infection is not uniformly fatal.

*The type of poultry production is also critical to assess the risk of exposure. Birds kept for longer periods (laying hens, breeder birds and slow-growth meat birds) have a longer duration of potential exposure to AI virus than short-lived poultry (broiler-type meat birds).  *

- Also important is the level of biosecurity in the premises in which birds are reared. Long-lived breeder birds tend to be a lower risk because of the higher biosecurity measures in place. It is easier to obtain high level immunity in long lived meat birds than in white feathered broilers given the difficulty in giving 2 doses of vaccine during the short life span of the latter.

*In addition to being considered as genetically modified organisms, the potential of live ND vectored vaccines to spread to non-target species and to unvaccinated flocks often raises concerns during the regulatory review and vaccine licensing process*

- Often?
- Coverage of the ND vector vaccines seems disproportionate compared to other vaccines given they have not been widely deployed in most parts of the world.

*There are several new experimental AI vaccine approaches not currently licensed for commercial use including wild type or attenuated LPAI; use of various vectors e.g. adenovirus, salmonella, avian leukosis and vaccinia; eukaryotic systems e.g. plants; and DNA vaccines, with a main objective being a universal vaccine covering against all subtypes of HPAI.*

- Other developments include improved adjuvants/immunostimulants (e.g. Toll-like receptor agonists, splicing to flagellar proteins etc.) and inclusion of M2e protein to broaden protection  (see, for example, Li *et al.*, 2020 ^4^ **, **Lin *et al.*, 2020 ^5^). You should also refer to duck virus enteritis vector vaccines because these potentially offer protection against another important viral disease that causes high mortality in ducks and hence give greater reason for vaccinating ducks (Chen et al., 2019 ^6^).  
- I recommend splitting this section in two given most of these vaccines are not aiming to be universal. It would also be worth commenting more about mucosal immunity and cell mediated immunity – see Wang *et al.* (2020 ^7^).

*Review of the vaccine database from The Center for Food Security and Public Health, Iowa State University showed that of 43 manufacturers listing vaccines for the control of Avian Influenza, 31 (72%) are currently manufacturing vaccines combining AI with other antigens (CFSPH, 2018). *

- CFSPH is no longer providing this service and the link is broken. You can still point out that this was the situation several years ago. Across the paper much of the data are dated.  

*I* *rrespective of the vaccination schedule or the specific vaccines used, Tarigan et al. (2018)* *demonstrated that the birds were not protected throughout production. Without vaccination after 19 weeks, the birds failed to be protected after 38 weeks of age; with vaccination after 19 weeks, the birds failed to be protected around 58 weeks of age. The data clearly demonstrated showed that with laying or breeder birds the duration of immunity is one of the main limitation issues for life cycle protection in the field, especially in the smallholder setting.*

- The paper by Tarigan *et al.* was an important reminder that killed antigen vaccines may not give life-long immunity. However, a number of the farms using vaccine did not follow the recommendations of vaccine manufacturers in terms of dose schedules, providing some explanation for the poor results. One farm that did still had acceptable levels of antibody at 68 weeks.

*Due to the antigenic drift of the AI virus in the field, there is a need to constantly update the composition of AI vaccines.*

- This is true for killed antigen vaccines and some vector vaccines, but some vector vaccines have shown good cross clade protection (e.g. HVT-AI).

*Vaccination is expensive due to the need to conduct high quality post-vaccination surveillance to monitor the genetic and antigenic characteristics of circulating field viruses.*

- The cost of post-vaccination surveillance depends on the objectives of the surveillance program. If vaccinating in a country with endemic infection where the goal is to reduce the effects of the disease and levels of circulating virus then the main objective is to monitor for antigenic variant viruses. This is a small cost compared to the cost of a large-scale campaign.

*Poor vaccination practices, insufficient vaccination coverage and lack of post-vaccination monitoring (as often seen in developing countries with large backyard poultry populations) can contribute to an endemic situation*

- This section of the document does not focus sufficiently on any of the potential positive aspects of vaccination. It is reasonable to suggest that vaccination of backyard poultry is difficult, but it also depends on the quality of services available to small scale producers. For example, in border areas in China adjacent to Vietnam all poultry are vaccinated on a regular basis to develop an “immune” buffer zone.
- Table 2 - I am not sure of the need for Table 2. I would recommend rechecking the data for Asia and the Americas and whether these are deaths directly from HPAI or deaths plus deaths from culling. If the latter, then the number for the Americas is too low.
- Table 3 does not contain multiple antigens introduced recently – now up to Re-11 and Re-12 for H5 vaccines. Three H7N9 antigens have been introduced – the last one Re-3 only a few weeks ago  
- The most common bivalent or trivalent vaccine against HPAI is H5/H7 vaccine used in China.

**Table 5:**

- There has been consensus for a number of years that an improved vaccine specifically for ducks that also addresses another disease (DVE) would be preferable. One of the vaccines that does offer broad cross clade protection in chickens (HVT-AI) does not protect domestic ducks.
- The OFFLU Beijing meeting concluded “Any country considering vaccination of household poultry should take into account the difficulties that have been encountered in implementing and sustaining these programs in some countries. Intensive programs have been implemented successfully for limited time period in some countries where public health was a priority.” This should be included in the paper. 

**On universal vaccines:**

*The antigenic drift of the AI viruses in the field requires a constant surveillance usually poorly performed by national veterinary services in developing countries due to the lack of proper facilities, human and financial resources. In addition, the availability of universal vaccines would eliminate the need to regularly update vaccines, often affected by inefficient national regulatory review and approval processes for vaccines*

- This does not reflect the work that has been done in Vietnam, Indonesia, China and Egypt to monitor antigenic change and where new vaccine antigens have been introduced in response to antigenic drift or introduction of a new antigenic variant (e.g. clade 2.3.4.4b to Egypt). A universal vaccine might be feasible, but it is unlikely that this would be a single dose vaccine. HVT-AI vector vaccine meets some of these requirements.

*Such a vaccine would be also available in countries considering adding vaccination to their AI control measures even though the current trend shows that fewer countries are willing to initiate AI vaccination.*

- Vaccination stockpiles are now in place in several countries including the US and the Republic of Korea, indicating these could be used in the event of an H5Nx outbreak. These are both places that previously had not vaccinated against HPAI. Iran is also using vaccine against H5 avian influenza (Baradaran Seyed *et al.*, 2019 ^8^).

**Conclusion:**

*Vaccination against HPAI has been used as one of the tools in national AI control programmes with varying degrees of successes *

- Measuring success depends on the objectives of the vaccination programme. In places like Vietnam the main reason for introducing vaccination was to reduce the number of human infections and to move away from culling millions of birds (many owned by smallholders) given this had not resulted in elimination of the virus. Similar for H7N9 vaccination in China. In none of the “endemic” countries was the goal elimination of virus.

*Few countries have been able to eradicate HPAI and these countries have typically relied on extremely competent national veterinary services and / or had limited poultry population at risk. *

- Only true if you are referring to places that have used vaccination as part of an elimination programme.

*The cost of AI vaccines is only a small part of the overall cost of vaccinating poultry against AI, especially when targeting poultry smallholders in lower- and middle-income countries. The current vaccine production technologies and vaccines have not been able to address the cost inherent to their use.*

- Figures in your table 5 suggest otherwise in that the cost of administering the vaccine is lower than the cost of administration. See previous discussion on surveillance if the goal is not virus elimination.

*In order to represent a significant advance, the profile for vaccine candidates must include protection against all viral strains (universal vaccine), efficacy in ducks with reduction in viral shedding and, protection after a single administration. *

- Universal vaccines tend be aimed at all strains of Influenza A virus. A duck specific virus that generates strong immunity would still be valuable. Single dose vaccination is already in use in ring vaccination around outbreaks.

*Without the inclusion of these properties in the vaccine profile, it is unlikely that any vaccine candidate would add significant value to the current AI vaccine market and thus warrant the investment of funds for its development.* *In order to represent a significant advance, the profile for vaccine candidates must include protection against all viral strains (universal vaccine), efficacy in ducks with reduction in viral shedding and, protection after a single administration. Without the inclusion of these properties in the vaccine profile, it is unlikely that any vaccine candidate would add significant value to the current AI vaccine market and thus warrant the investment of funds for its development.*

- Existing vaccines can still provide protection against disease and reduce shedding. The logical approach to duck vaccination is to use a vector vaccine that also provides protection against another pathogen such as duck virus enteritis vaccine.
- Poultry vaccines will not be developed specifically for the smallholder market. Many smallholder birds are not vaccinated for other important endemic diseases.
- Some registered vaccines that afford good cross protection are available (see for example Oliveira Cavalcanti *et al.*, 2017 ^9^ and Nassif *et al.*, 2020 ^10^). HVT vector can only be used in well-managed hatcheries.

**Additional references that could be included:**

1. Delabouglise *et al.* (2020 ^1^).
2. Wang *et al.* (2014 ^2^).
3. King *et al.* (2020 ^3^).
4. Li *et al.* (2020 ^4^).
5. Lin *et al.* (2020 ^5^).
6. Chen *et al. *(2019 ^6^).
7. Wang *et al. *(2020 ^7^).
8. Baradaran Seyed *et al.* (2019 ^8^).
9. Oliveira Cavalcanti *et al.* (2017 ^9^).
10. Nassif *et al.* **(2020 ^10^). 

Does the article adequately reference differing views and opinions?

Partly

Are all factual statements correct, and are statements and arguments made adequately supported by citations?

Partly

Is the Open Letter written in accessible language?

Yes

Where applicable, are recommendations and next steps explained clearly for others to follow?

Partly

Is the rationale for the Open Letter provided in sufficient detail?

Yes

Reviewer Expertise:

I am an animal disease manager with over 25 years experience in the control and prevention of avian influenza, including use of vaccination.

I confirm that I have read this submission and believe that I have an appropriate level of expertise to confirm that it is of an acceptable scientific standard, however I have significant reservations, as outlined above.

## References

[1] : Poultry farmer response to disease outbreaks in smallholder farming systems in southern Vietnam. *eLife*.2020;9: 10.7554/eLife.59212 10.7554/eLife.59212 PMC750565432840482

[2] : H6 influenza viruses pose a potential threat to human health. *J Virol*.2014;88(8) : 10.1128/JVI.03292-13 3953-64 10.1128/JVI.03292-13 24501418PMC3993743

[3] : The genetics of highly pathogenic avian influenza viruses of subtype H5 in Germany, 2006-2020. *Transbound Emerg Dis*.2020; 10.1111/tbed.13843 10.1111/tbed.13843 32964686

[4] : Lactobacillus plantarum surface-displayed influenza antigens (NP-M2) with FliC flagellin stimulate generally protective immune responses against H9N2 influenza subtypes in chickens. *Vet Microbiol*.2020;249: 10.1016/j.vetmic.2020.108834 108834 10.1016/j.vetmic.2020.108834 32919197

[5] : Induction of Robust Immune Responses by CpG-ODN﻿-Loaded Hollow Polymeric Nanoparticles for Antiviral and Vaccine Applications in Chickens. *Int J Nanomedicine*.2020;15: 10.2147/IJN.S241492 3303-3318 10.2147/IJN.S241492 32494131PMC7227821

[6] : Protective efficacy in farmed ducks of a duck enteritis virus-vectored vaccine against H5N1, H5N6, and H5N8 avian influenza viruses. *Vaccine*.2019;37(40) : 10.1016/j.vaccine.2019.08.026 5925-5929 10.1016/j.vaccine.2019.08.026 31471151

[7] : Emerging Role of Mucosal Vaccine in Preventing Infection with Avian Influenza A Viruses. *Viruses*.2020;12(8) : 10.3390/v12080862 10.3390/v12080862 PMC747210332784697

[8] : Control of Highly Pathogenic Avian Influenza by Evidence-Based Vaccinology: Past Progress, Future Prospect. *Iranian Journal of Epidemiology*.2019;15(1) :77-86 Reference source

[9] : A genetically engineered H5 protein expressed in insect cells confers protection against different clades of H5N1 highly pathogenic avian influenza viruses in chickens. *Avian Pathol*.2017;46(2) : 10.1080/03079457.2016.1250866 224-233 10.1080/03079457.2016.1250866 27807985

[10] : Herpesvirus of turkey-vectored avian influenza vaccine offers cross-protection against antigenically drifted H5Nx highly pathogenic avian influenza virus strains. *Avian Pathol*.2020; 10.1080/03079457.2020.1790502 1-10 10.1080/03079457.2020.1790502 32615785

# Peer review: Reviewer response for version 1

**DOI:** 10.21956/gatesopenres.14370.r29690

**Published:** 2020-10-21

**Authors:** Erica Spackman

**Affiliations:** 1 Southeast Poultry Research Laboratory, United States Department of Agriculture (USDA)-Agricultural Research Service (ARS), Athens, GA, USA

Although I generally agree with the conclusions, the authors seem to lean more “all-or-nothing” than I do when it comes to improvements in vaccines; incremental improvements would likely be beneficial. Also, if commercial poultry are being vaccinated in a region experiencing an AIV outbreak, including back yard poultry in vaccination efforts may be beneficial to food security, economic stability, and disease control (provision of resources by the government would probably be necessary in many situations). All ideal features listed for vaccines for backyard poultry would greatly benefit commercial poultry vaccination as well. Developing vaccines that fulfill these criteria will be difficult; capitalizing on any single improvement should be considered within the scope of a given situation. In general, this is a very good, succinct summary of the topic. A few comments on some statements from the letter are listed below add clarity and come additional context.

**General:**

- How is “backyard/small holder” poultry defined in the letter? FAO sectors 3 and 4 may be appropriate.

 

**Introduction:**

- “...Indonesia and Vietnam) where massive vaccination campaigns have been initiated since 2004 (accounting for more than 99% of the use of AI vaccines)…” - Note that HPAIV was probably already endemic when vaccination was initiated.

 

**AI – etiology and epidemiology:**

- “…16 H strains and 9 N strains…” -The H and N refer to subtypes, not strains.
- “AI viruses in poultry are classified as either low pathogenic (LPAI) or highly pathogenic (HPAI)…” - Importantly, this applies to gallinaceous poultry (the testing is done in chickens and ducks are considered poultry but as stated by the authors, they are often silent carriers even for strains that are deadly for chickens).
- “H5N1 HPAI viruses have been sub-classified according to Clades, or…” – The clade system for the goose/Guangdong/1996 lineage refers to the H5 HA only, the NA subtypes and lineages vary.

 

**AI vaccines and vaccination:**

- “…3) HA DNA vaccine adjuvanted;…” - Although licensed in the US this vaccine platform has not been utilized in the field (as of C. 2020).
- Need context as to why the NDV vector is the focus. It’s not widely used in the field because of interference from immunity to the vector as discussed. Of the vectored vaccines, HVT and Fowlpox are most common.
- “…rHVTAIV has the advantage that it can be used in day-old chicks at the hatchery.” - And can be also be administered *in ovo*.
- “Vaccination with high-quality, registered vaccines, according to established protocols reduces resistance to infection…” - Should this be “increases” resistance? There is not much work on vaccination and infectious dose for AIV, what data are available (for inactivated vaccines) suggest that vaccination doesn't affect the infectious dose, which is consistent with how the vaccine induces immunity.  
- “Vaccination is expensive due to the need to conduct high quality post-vaccination surveillance to monitor the genetic and antigenic characteristics of circulating field viruses.”  - This is true of long-term vaccination, if the outbreak can be halted vaccine use may be stopped. However, surveillance may be conducted regardless of vaccination.
- “As a rule, it is not recommended to vaccinate backyard poultry since maintaining adequate levels of immunity is extremely difficult.” - Alternatively, if the birds have some immunity it might contribute to better food security and cost-benefit by improving survival and maintaining production if there is a high level of virus challenge in the field. 
- Table 5: “Mass application” - The only AIV vaccine which might be given by spray application are avian paramyxovirus type 1 (NDV) vaccine vectored products which is a very small proportion of vaccines that have been utilized (i.e. well below 4.5%). 
- Tables 5 and 6: “Use in multiple species” - When they are tested, the current inactivated vaccines usually work well in ducks. However, data on vaccines in ducks and some poultry species are lacking. Vectored vaccines are restricted to the hosts of the vector.

Does the article adequately reference differing views and opinions?

Yes

Are all factual statements correct, and are statements and arguments made adequately supported by citations?

Partly

Is the Open Letter written in accessible language?

Yes

Where applicable, are recommendations and next steps explained clearly for others to follow?

Not applicable

Is the rationale for the Open Letter provided in sufficient detail?

Yes

Reviewer Expertise:

My areas of expertise are avian influenza virus vaccines, diagnostics and pathobiology.

I confirm that I have read this submission and believe that I have an appropriate level of expertise to confirm that it is of an acceptable scientific standard.

# Peer review: Reviewer response for version 1

**DOI:** 10.21956/gatesopenres.14370.r29689

**Published:** 2020-10-21

**Authors:** Gavin J.D. Smith

**Affiliations:** 1 Programme in Emerging Infectious Diseases, Duke-NUS Medical School, Singapore, Singapore

Here the authors have provided a nice overview of the challenges in controlling highly pathogenic avian influenza, with a focus on the challenges and limitations of vaccination as a control measure. There is also a discussion of how vaccines and vaccination could be improved to help small holders, who are proportionately more impacted by HPAI outbreaks. Overall I find this an interesting discussion and a worthwhile contribution to the area, but there are some inaccuracies that should be addressed prior to indexing.

 Primarily the authors are incorrect in assuming that control AI in poultry was the main driving force for the use of vaccines. Thailand did not vaccinate because they are a major poultry exporter and use of vaccines would jeopardise the ability to certify poultry was free of AI. So they used testing and culling. In Hong Kong there was a combination of measures used, with mass culling and testing regimes also implemented. Other countries used vaccination not just to reduce poultry death but to reduce overall levels of virus in poultry which leads to less human infection. It would therefore be helpful to have a more detailed discussion of the uses of vaccination in combination with other control measures.

 And it is not just vaccination that drove viruses to become endemic in poultry. The presence of ducks, which are largely immune to HPAI and are frequently asymptomatically infected, is an important factor, particularly as vaccine are largely ineffective in them. There were also long delays in implementing any effective control measures in many countries, providing time for the virus to establish in poultry and frustrating control measures. Poor biosecurity can also contribute to endemicity.

 Minor points:

1. Paragraph 2 (Para 2) there is also influenza D.
2. Para 3, enveloped or not is not relevant here. Also info on the number of HA and NA types is dated.
3. Is Table 1 correct, e.g. is the Oceanic H7N2 truly HPAI?
4. Para 8 starting with *“The type of poultry production is also critical to assess the risk of exposure”* needs some references.

Does the article adequately reference differing views and opinions?

Partly

Are all factual statements correct, and are statements and arguments made adequately supported by citations?

Partly

Is the Open Letter written in accessible language?

Yes

Where applicable, are recommendations and next steps explained clearly for others to follow?

Yes

Is the rationale for the Open Letter provided in sufficient detail?

Yes

Reviewer Expertise:

Virus evolution, AI molecular epidemiology, vaccine strain selection.

I confirm that I have read this submission and believe that I have an appropriate level of expertise to confirm that it is of an acceptable scientific standard, however I have significant reservations, as outlined above.

# Peer review: Reviewer response for version 1

**DOI:** 10.21956/gatesopenres.14370.r29700

**Published:** 2020-10-12

**Authors:** Luca Busani

**Affiliations:** 1 Dipartimento di Malattie Infettive, Istituto Superiore di Sanità, Rome, Italy

In the manuscript “Are current avian influenza vaccines a solution for smallholder poultry farmers?” the authors present the situation of the Highly pathogenic avian influenza, a list of limiting factors for the vaccination in smallholder settings and their opinion on what the characteristics of the ideal vaccine to target the poultry smallholder segment should have.

 The paper is presented as an overview, without any kind of analysis or original data presented.

 The paper is well written and the items are clearly presented, but I would suggest to the authors to add some details about the focus of the manuscript, that is the smallholder poultry farms: in particular a clear definition (FAO presents some descriptions in the Chapter 1 “The socio-economic Importance of Family Poultry” http://www.fao.org/3/y5169e/y5169e02.htm#TopOfPage) and descriptions (backyard flocks are often multispecies and multiage, with poor or no biosecurity measures, a complex setting for vaccination). This is in my opinion important, to give to the reader a clear picture of the challenges of vaccinating smallholder flock.

 Moreover, the background presents information on avian influenza epidemics and vaccine schedules, but mainly for commercial poultry. Additional comments on the impact of the AI epidemics on smallholders and examples of vaccination in backyard flocks should be provided as comparison (Individual and flock immunity responses of naïve ducks on smallholder farms after vaccination with H5N1 Avian Influenza vaccine: a study in a province of the Mekong Delta, Vietnam (Huynh *et al.*, 2019 ^1^).

 Among the positive key factors for the successful control of AI in poultry, the veterinary services capacity is mentioned, but I would also suggest to add the need of lab capacity for diagnosis of poultry diseases and the regular use of the lab by vet public services, whenever possible.

 In table 6, at the universal vaccine rationale a possible interpretation is if such universal vaccine would become available, also the surveillance could be avoided. I would suggest to clarify this sentence pointing out the need of basic surveillance to ensure detection of the circulating viruses and vaccination failures.

 In table 6, “use in multiple species” I would also suggest to add “multiple ages”.

Does the article adequately reference differing views and opinions?

Partly

Are all factual statements correct, and are statements and arguments made adequately supported by citations?

Partly

Is the Open Letter written in accessible language?

Yes

Where applicable, are recommendations and next steps explained clearly for others to follow?

Yes

Is the rationale for the Open Letter provided in sufficient detail?

Yes

Reviewer Expertise:

Public Health, infectious diseases, zoonoses

I confirm that I have read this submission and believe that I have an appropriate level of expertise to confirm that it is of an acceptable scientific standard, however I have significant reservations, as outlined above.

## References

[1] : Individual and flock immunity responses of naïve ducks on smallholder farms after vaccination with H5N1 Avian Influenza vaccine: a study in a province of the Mekong Delta, Vietnam. *PeerJ*.2019;7: 10.7717/peerj.6268 e6268 10.7717/peerj.6268 30671307PMC6339474
